# Seqinspector: position-based navigation through the ChIP-seq data landscape to identify gene expression regulators

**DOI:** 10.1186/s12859-016-0938-4

**Published:** 2016-02-12

**Authors:** Marcin Piechota, Michal Korostynski, Joanna Ficek, Andrzej Tomski, Ryszard Przewlocki

**Affiliations:** Department of Molecular Neuropharmacology, Institute of Pharmacology Polish Academy of Sciences, Krakow, 31-344 Poland

**Keywords:** ChIP-seq, RNA-seq, Microarray, Gene expression, Promoter analysis, Transcription factor

## Abstract

**Background:**

The regulation of gene expression in eukaryotic cells is a complex process that involves epigenetic modifications and the interaction of DNA with multiple transcription factors. This process can be studied with unprecedented sensitivity using a combination of chromatin immunoprecipitation and next-generation DNA sequencing (ChIP-seq). Available ChIP-seq data can be further utilized to interpret new gene expression profiling experiments.

**Results:**

Here, we describe seqinspector, a tool that accepts any set of genomic coordinates from ChIP-seq or RNA-seq studies to identify shared transcriptional regulators. The presented web resource includes a large collection of publicly available ChIP-seq and RNA-seq experiments (>1300 tracks) performed on transcription factors, histone modifications, RNA polymerases, enhancers and insulators in humans and mice. Over-representation is calculated based on the coverage computed directly from indexed files storing ChIP-seq data (bigwig). Therefore, seqinspector is not limited to pre-computed sets of gene promoters.

**Conclusion:**

The tool can be used to identify common gene expression regulators for sets of co-expressed transcripts (including miRNAs, lncRNAs or any novel unannotated RNAs) or for sets of ChIP-seq peaks to identify putative protein-protein interactions or transcriptional co-factors. The tool is available at http://seqinspector.cremag.org.

**Electronic supplementary material:**

The online version of this article (doi:10.1186/s12859-016-0938-4) contains supplementary material, which is available to authorized users.

## Background

The regulation of gene expression is one of the most complex processes in living organisms [1]. This process is a result of interactions between core transcription factors, additional regulators and chromatin architecture [2]. Coupling chromatin immunoprecipitation with massive parallel sequencing (ChIP-seq) has provided a breakthrough in the analysis of gene-expression machinery. ChIP-seq data released by the Encyclopedia of DNA Elements consortium (ENCODE) [3] and other researchers have made it possible to analyze the functional regulatory genome on an unprecedented scale. The utilization of available ChIP-seq results can provide insights into multiple lines of investigation, including the search for molecular factors involved in the control of coordinated expression of a particular set of genes as well as for protein-protein interactions within peaks resulting from new ChIP-seq experiments. Such analyses require statistically oriented software to handle large genomic data in a computationally efficient manner.

Here, we present seqinspector, a bioinformatics service that utilizes up-to-date ChIP-seq data. Our tool allows the functional enrichment of user-defined lists of genes, transcripts and ChIP-seq peaks. The user is not limited to a predefined set of promoter regions and can use any set of genomic coordinates. Our tool provides solutions for multiple challenges that have arisen with new sequencing technologies. The first difficulty encountered in interpreting RNA-seq experimental results is that the output encodes multiple novel noncoding or un-annotated transcripts that are not represented in current databases. Another challenge that is often faced is the compatibility of the tool with dynamic and continuously updated genome annotations. Additionally, the tool should provide users the opportunity to search for protein binding sites in regions outside of gene promoters. For example, users might want to search for specific proteins that bind to the introns of differentially spliced genes or analyze peak regions obtained from new ChIP-seq experiments. Therefore, we eschewed the standard approach that uses gene annotations directly or converts genomic coordinates to gene annotations. Instead, our tool converts gene annotations into genomic ranges. For this purpose, we developed a new strategy for data storage, raw genomic bigwig tracks instead of an SQL system. These tracks are analyzed every time the user inputs a query. This method allowed us to develop a new coverage-based approach to calculate enrichment statistics.

## Implementation

### Storage of large collections of raw genomic files

Next-generation sequencing generates vast amounts of data. The results are typically presented as signal information (a per-base estimate across the genome) and as discrete elements (regions computationally identified as enriched from the signal). The most useful file formats for storing ChIP-seq experimental results are bigwig and bigbed for signal information and discrete elements (peaks), respectively [4]. Both formats are binary and indexed, and both can be used in the same way as tables in database systems. Therefore, we developed a database core using the bigwig file format instead of the SQL database system. We used a client based on the source code from the Integrative Genomics Viewer (IGV) genome browser to navigate bigwig files [5]. This approach, if parallelized, allows one to browse through multiple coverage tracks in a relatively short amount of time. Moreover, this strategy allows us to utilize raw data downloaded from various resources without requiring data transformation or reformatting. At the time of writing this manuscript, the data collection contains 463 tracks from mice and 697 tracks from humans. These data have been downloaded from various sources including ENCODE [3], FANTOM5 [6] (345 tracks), Gene Expression Omnibus (GEO) [7] (113 tracks) and the Short Read Archive (SRA) (11 tracks).

### A statistical test for the over-representation of ChIP-seq defined DNA features

The decision to choose a storage method between raw coverage (bigwig files) and peaks derived from ChIP-seq data (bigbed files) was critical to further implementing the system. Despite being computationally challenging, raw coverage files were favored because they can be directly used in visual explorations. Therefore, we used raw coverage files to store the data. This process required a different statistical approach than commonly implemented in over-representation analyses that assume a hypergeometric data distribution [8–10]. We decided to use a parametric statistical test that allows a comparison between the coverage means (length-normalized and log-transformed) from genomic coordinates between different samples. We implemented a two-sample *t*-test to compare the ChIP-seq track coverages derived from two samples of genomic ranges that were provided by the user and the background set. By default, the background set consists of 1000 random gene promoters and can be replaced by the user-defined set. A statistic based on the parametric z-score is used for a single-gene exploration.

### Seqinspector user interface

**Fig. 1:**
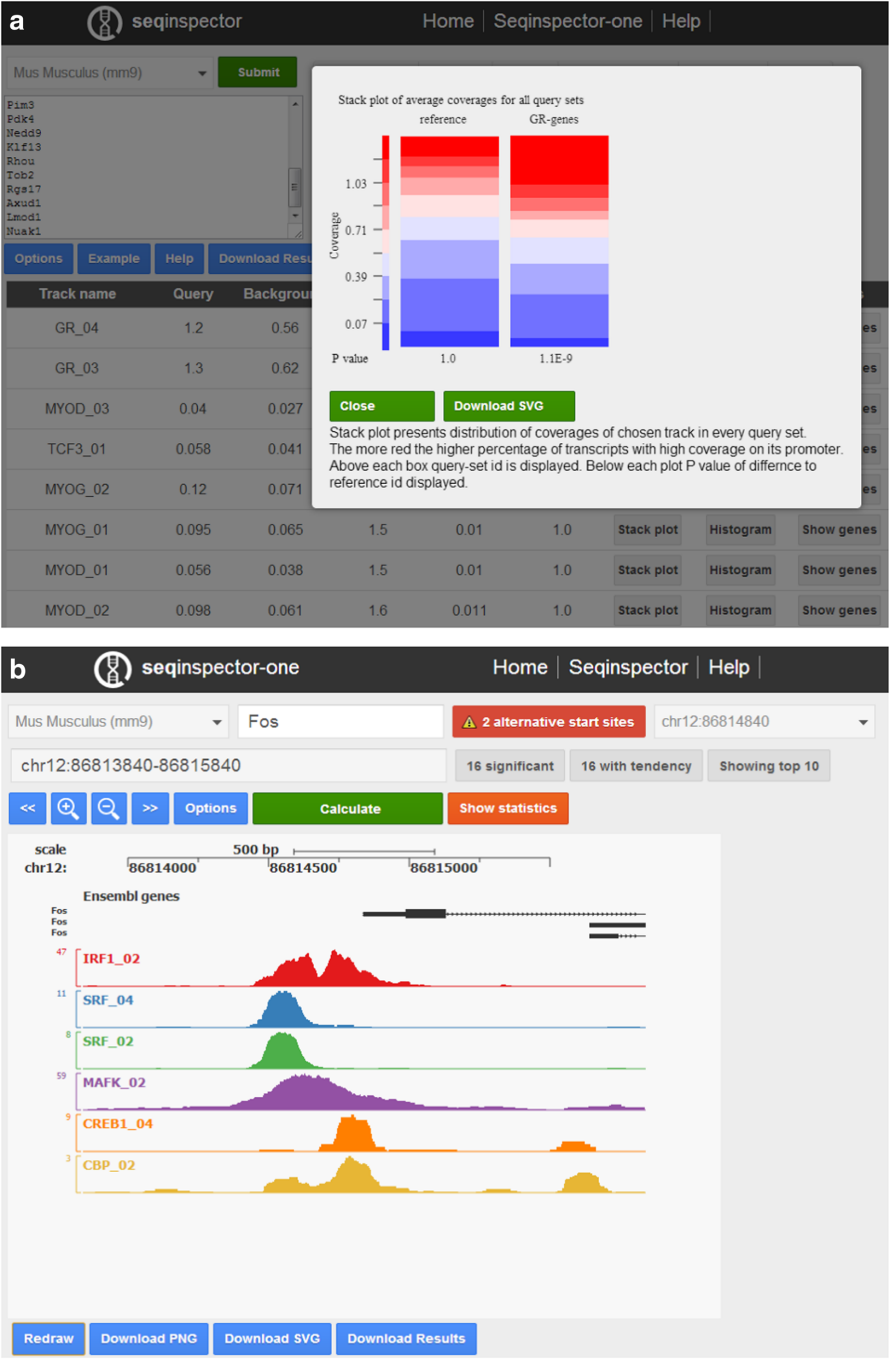
**a** Output of seqinspector showing the list of dexamethasone-regulated genes (input box on the upper-left), over-represented ChIP-seq tracks (*bottom-left*) and a stack plot with the distribution of coverages in the reference and query sets. **b** Output of a single-gene inspection showing the ChIP-seq tracks over-represented at the Fos gene promoter

Based on previous experience with developing user interfaces for bioinformatics resources [11, 12], we decided to minimize the number of possible options. The user must select the genome assembly (between *Mus musculus* mm9 and mm10 and *Homo sapiens* hg19) and the query input (Fig. 1a). The server automatically recognizes the type and identification format. Some of the most popular identification types are acceptable (Ensembl transcript ID, RefSeq mRNA and gene symbol). The user can also input a genomic range in both bed and genomic coordinate formats. Moreover, it is possible to input multiple gene sets individually and use one of them as the background. The selected gene lists can be compared with the background set using the “Statistics” button. As a result, the user will obtain a list of ChIP-seq tracks sorted by statistical enrichment. The following columns are present in the output table: (1) Track name—internal identifier of the track containing a short name of the transcription factor; (2) Query—average coverage in the query set; (3) Background—average coverage of the reference set; (4) Fold difference—fold difference between the query and reference; (5) *P*-value—the significance of the difference between the query and reference datasets (calculated by *t*-test); (6) Bonferroni—Bonferroni-corrected *P*-value; (7) Stack plot—stacked plots presenting the distribution of coverages in all query sets with respective *p*-values; (8) Histogram– a visualization of the average coverage (2000 bp around the center of the genomic interval) for all query sets; (9) Genes—genomic intervals in the query set, symbols for nearest genes and coverage for these intervals; and (10) Description—description of the track.

## Results

### Common regulators of co-expressed genes

seqinspector can be used for different types of analyses. One is to study clusters of co-expressed genes to find their putative molecular regulators. To demonstrate this functionality, we utilized the results from gene expression profiling of mouse astroglial primary cultures treated with dexamethasone [13]. Dexamethasone is an agonist of a nuclear transcription factor, the glucocorticoid receptor (GR). The list of 24 gene symbols for dexamethasone-regulated transcripts was submitted to seqinspector (seqinspector.cremag.org) (see Additional file 1). The genome assembly was set as *Mus musculus* (mm9), and the default background was used. seqinterpreter correctly identified GR (*P* = 0.0024; Bonferroni corrected) as a true-positive regulator of the genes from the list (see Table 1) and provided a profile of the GR binding sites for each analyzed gene.

**Table 1:** Top five enriched ChIP-seq tracks in the promoters of genes regulated by dexamethasone

Track ID	Transcription factor	Query coverage	Background coverage	*P*-value	Bonferroni-corrected *P*-value
GR_04	Glucocorticoid receptor	1.3	0.59	6.7 × 10^-6^	**0.0024**
GR_03	Glucocorticoid receptor	1.3	0.64	2.2 × 10^-5^	**0.008**
MYOG_01	Myogenin	0.096	0.059	0.0017	0.59
TCF3_01	Transcription factor 3	0.058	0.038	0.0018	0.63
FOSL1_01	Fos-related antigen 1	0.021	0.016	0.0093	1.0

Only the over-representation of GR-tracks was found statistically significant (Bonferroni corrected *P* < 0.05, typed in bold)

### Protein-protein interactions

seqinterpreter can be applied to study protein-protein interactions. seqinterpreter calculates the average coverage for all stored ChIP-seq tracks for the submitted genomic ranges. The obtained coverages are then compared with a reference dataset using a two-sample *t*-test followed by correction for multiple testing. To demonstrate this functionality, we utilized data from a ChIP-seq analysis of SP1 binding in GM12878 human lymphoblastoid cells (data available at GEO GSM803363). We submitted the top 358 peaks (>3000 signal value) from this dataset to seqinterpreter as genomic ranges in bed format (Additional file 2). We submitted the lowest 358 peaks as background. We used the *Homo sapiens* hg19 assembly. The transcription factors ATF3, SP2, NFYA, NFYB, E2F4, IRF1 and SRF indirectly bind to SP1 binding sites [14]. seqinspector correctly identified the enrichment of the following factors as interacting proteins: (1) ATF3 (*P* = 0.013), (2) SP2 (*P* = 1.7 × 10^-33^), (3) NFYA (*P* = 8.9 × 10^-47^), (4) NFYB (*P* = 9.6 × 10^-41^), (5) E2F4 (*P* = 1.8 × 10^-16^), (6) IRF1 (*P* = 2.2 × 10^-7^) and (7) SRF (*P* = 1.2 × 10^-6^). The tool identified also other transcription factors binding to the same sites, including IRF3 (*P* = 3.0 × 10^-43^), C-FOS (*P* = 4.0 × 10^-42^) and CHD2 (*P* = 6.8 × 10^-41^). All of the presented *P*-values are Bonferroni corrected.

### Cell type enrichment

**Fig. 2:**
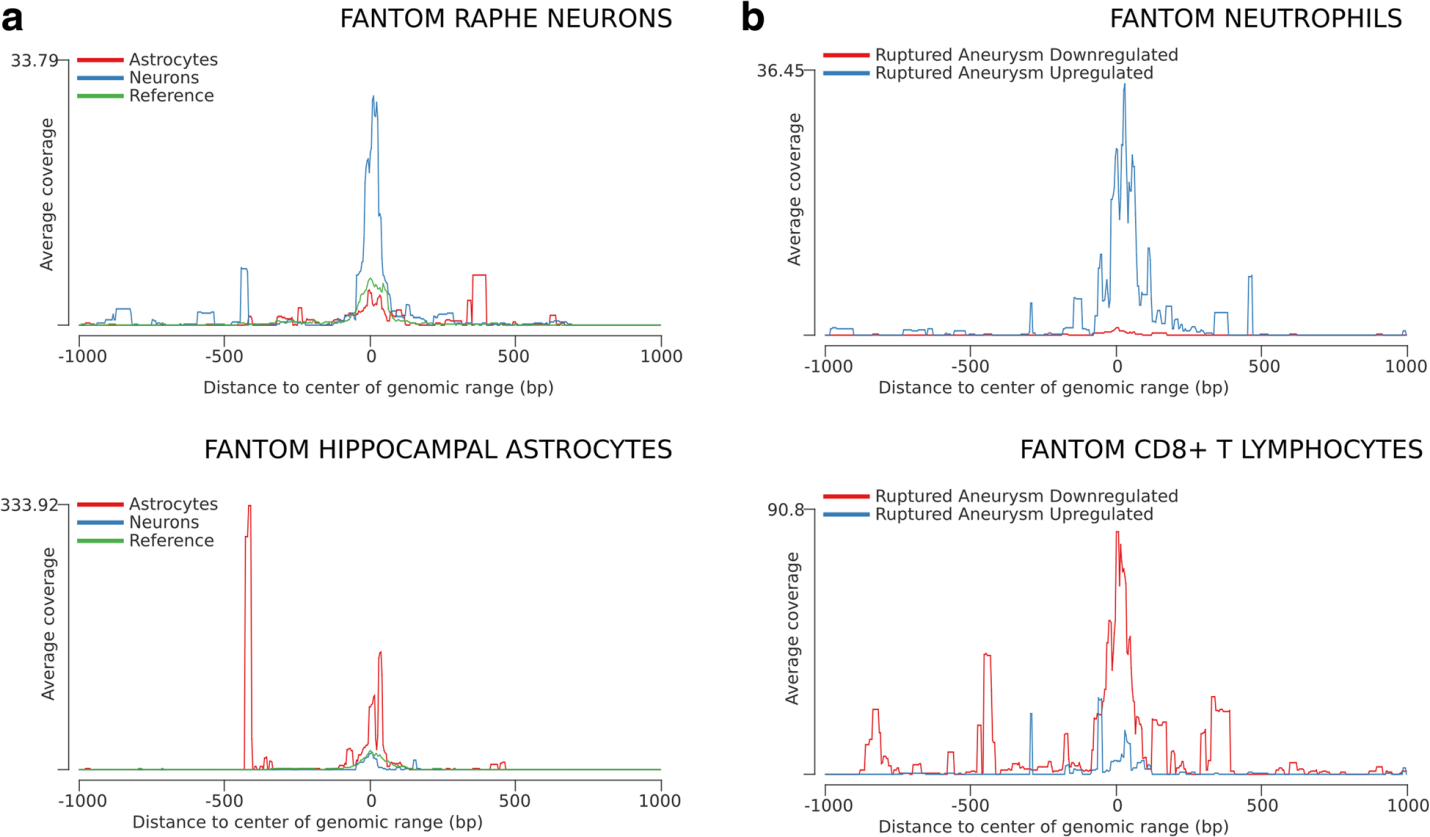
Identification of cell-type-specific transcript expression start site. The plots display the mean coverage for the selected gene sets in various cell types based on the CAGE data. The x-axis represents the genomic region around transcription start sites from 5’ to 3’. The y-axis represents the coverage that has been normalized to the number of aligned tags per million. **a** Coverage histograms for neuron-specific (*blue*) and astrocyte-specific (*red*) gene lists from Zhang et al. [15]. The upper panel displays the mean coverage of raphe neurons CAGE tags, whereas the bottom histogram refers to the hippocampal astrocyte CAGE tags. **b** Coverage histograms for genes up-regulated (*blue*) and down-regulated (*red*) in ruptured intracranial aneurysm from Pera et al. [16]. The upper panel presents the mean coverage of neutrophil CAGE tags, whereas the bottom (average) histogram refers to CD8+ T lymphocytes CAGE tags

Another straightforward application of seqinspector is the study of transcript expression in various tissue and cell types. For this purpose, tracks generated by the FANTOM5 project using cap analysis of gene expression were added to the seqinspector database (264 tracks for human and 81 for mouse after manual curation). This type of analysis reveals active transcription start sites and gene variants expressed in particular cell populations. List of genes or transcripts derived from microarray or RNA-seq profiling experiments can be inspected for cell-type-specific gene expression. To provide an example of this utility, we used results of gene expression profiles in different cellular compartments of the nervous system [15]. Submission of neuron- and astrocyte-specific lists of genes (Additional file 3) confirmed cell-type enrichment and indicated which transcriptional start sites are utilized in these two cell populations (Fig. 2a). For astrocyte-specific genes, only one significantly over-represented track was noted - the Hippocampal Astrocytes CAGE track generated by the FANTOM5 consortium (CNhs12129.11709-123B8, *P* = 0.0034). For neuron-specific genes, 14 over-represented tracks were noted, including 13 FANTOM5 tracks generated from neural tissue or isolated neurons (e.g., Olfactory brain, CNhs10489.18-22I9, *P* = 2.4 × 10^-5^ and Raphe neurons, CNhs12631.11722-123D3, *P* = 3.1 × 10^-4^) and one ENCODE track for the neuron-restrictive silencer factor (NRSF, GSM915175, *P* = 0.026).

Another possible use of seqinspector is to estimate the distribution of transcriptional alterations among various cell populations, which may be estimated from results of gene expression profiling in a heterogeneous tissue. To demonstrate this functionality, we used a list of genes from expression profiling in whole-blood samples obtained from patients after ruptured intracranial aneurysms and a control group (Additional file 4) [16]. The lists of up-regulated and down-regulated genes were compared against each other. seqinspector identified over-representation of CD8+ T-lymphocyte-specific transcripts among the down-regulated genes (CNhs12178.12191.129B4, *P* = 0.005) and neutrophil-specific genes among the up-regulated genes (CNhs10862.11233.116C9, *P* = 0.12) (Fig. 2b). All of the presented *P*-values are Bonferroni corrected.

### Comparison with other tools

**Fig. 3:**
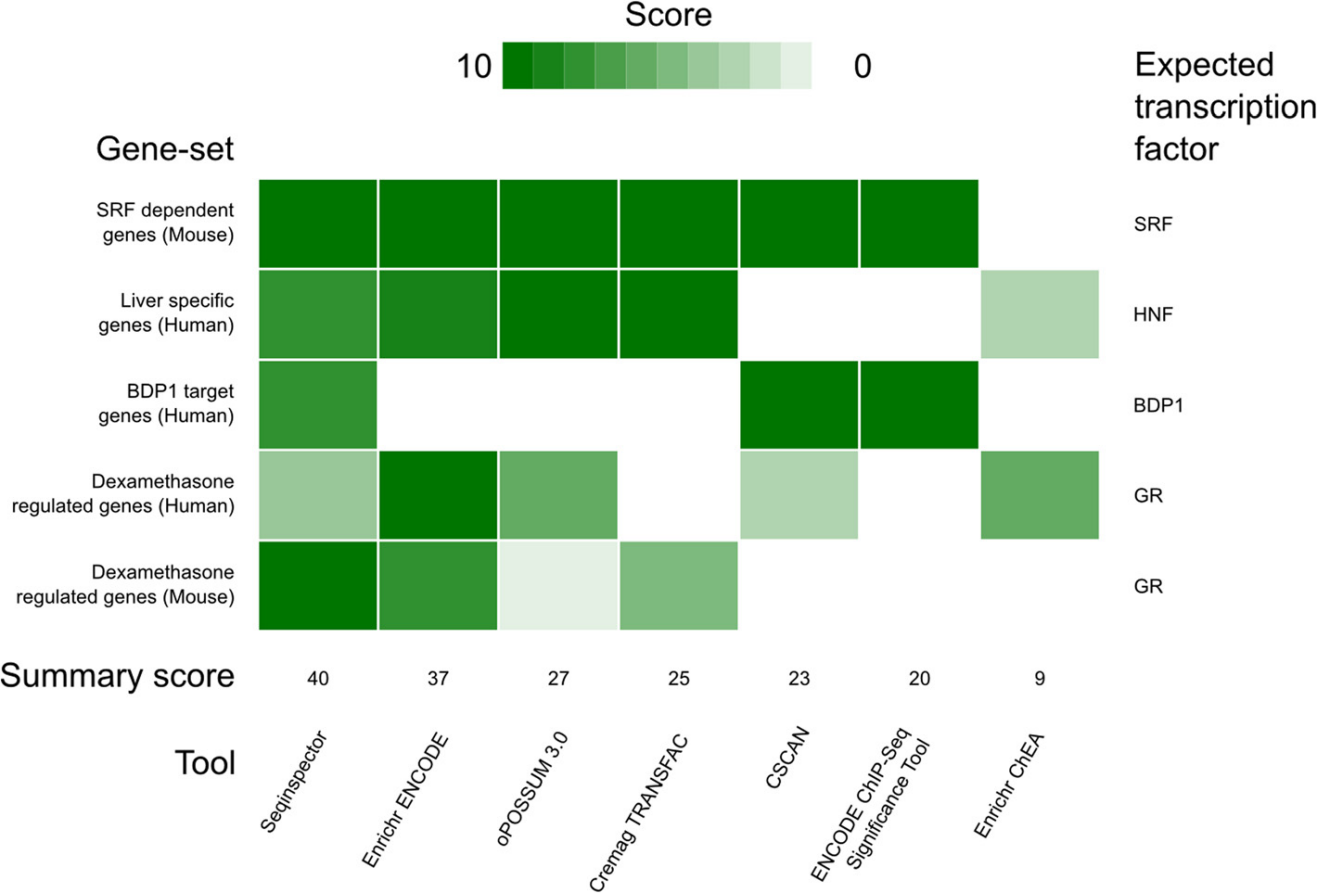
Comparison of seqinspector to other online tools. The heatmap presents the scores from seqinspector, Enrichr, oPOSSUM, Cremag, CSCAN and ENCODE ChIP-Seq Significance Tool (in columns) for five selected gene sets (in rows). The scores were calculated based on the rank in the results of the expected transcription factor (*on the right*) with ten points for first rank (*dark green color*), nine points for second and down to one point for the tenth rank (*white color*). The sum of the scores is presented at the bottom. The tools are ordered by the sum of their scores in decreasing order

To demonstrate the effectiveness of seqinspector, we compared this tool with available ChIP-seq data-based online tools (CSCAN [10], ENCODE ChIP-Seq Significance Tool [17] and Enrichr with ENCODE ChIP-seq and ChIP-x gene set libraries [18]) as well as tools based on *in silico* predicted transcription factor binding sites (oPOSSUM 3.0 [19] and Cremag [12]). For this purpose, we used the following five example sets of genes regulated by various transcriptional mechanisms. One from each tool excluding Enrichr (no list of genes with specified transcriptional factor was provided with this tool): (a) an example gene set provided in this paper—GR-dependent genes regulated in mouse; (b) a list of human genes regulated by dexamethasone from the ENCODE ChIP-Seq Significance Tool website; (c) BDP1 target gene set from the CSCAN website; (d) liver-specific gene set from the oPOSSUM 3.0 website and (e) a list of genes regulated by SRF from the Cremag website. We converted the original lists into Ensembl Transcript IDs and gene symbols using Biomart [20] to meet the tool-specific input requirements. We used the default settings for all of the tools for the comparison. As a score, we used the rank of the expected transcription factor in the obtained results, where the points one to ten were awarded with a maximum given for the first position on the list (Fig. 3). seqinspector received the highest summary score (score = 40) followed by Enrichr using ENCODE data (score = 37) and oPOSSUM 3.0 (score = 27). Thus, the seqinspector tool, which is based on parametric statistics for enrichment calculation, was comparable to other promoter analysis methods. All of the gene sets with the original IDs, RefSeq numbers, promoter sequences and results are provided in Additional file 5.

## Discussion

Comparative genomic studies suggest that 3 to 8 % of the sequence information in a genome is evolutionarily conserved among mammals [21]. Earlier *in silico* approaches to predict transcription factor binding sites were limited to those specific regions [22]. However, the ENCODE project found that the vast majority (~80 %) of the genome can be annotated with RNA- or chromatin-associated features (3). Therefore, the level of potentially functional genomic regions may be significantly higher than previously thought. seqinspector provides the possibility to search for functional elements with high confidence within unconserved genomic regions, offering a significant advantage over systems based on *in silico*-computed transcription factor binding sites. Another advantage of ChIP-seq–based enrichment services is the ability to search for distant enhancer transcription factors through indirect binding to promoter regions. Enhancer regions can be distant (even up to 200 kb) from the proximal promoter of a target gene [23]. To demonstrate this advantage, we assessed seqinspector using a set of genes regulated by the enhancer transcription factor GR.

A unique feature of seqinspector is that it accepts any set of genomic coordinates. Therefore, the tool can be used to identify common gene expression regulators for sets of co-expressed transcripts (including miRNAs, lncRNAs and any novel un-annotated RNAs) or for sets of ChIP-seq peaks to identify putative protein-protein interactions or transcriptional co-factors. Our software enables the study of transcript expression in various tissues and cell types using FANTOM5 CAGE data. Therefore, it is possible to identify the enrichment of cell-type-specific genes or transcripts. The seqinspector software package can also be used in more creative ways; for example, the user can compare the characteristics of 5’ untranslated region (UTR) histone modifications between two sets of transcripts. We are continuously adding new ChIP-seq experiments to the database. seqinspector is not easily outdated; any novel genomic features (e.g., genes, miRNAs or ChIP-seq peaks) can be used if their genomic positions are known. Even after major assembly changes in databases, the new genomic ranges can be translated to the old versions of the genome assembly using the liftover tool. This mechanism is currently built into seqinspector for the mouse mm10 assembly.

## Conclusions

The seqinspector tool was developed to facilitate the functional annotation and discovery of transcription factor binding sites on promoters of co-expressed transcripts, signals from ChIP-seq experiments and any other set of genomic coordinates sharing a common trait. The presented web resource includes a large collection of publicly available ChIP-seq experiments (>1100 tracks) performed on transcription factors, histone modifications, RNA polymerases and insulators in humans and mice.

### Availability and requirements

The seqinspector tool is free, open to all users, and there is no login requirement. seqinspector is available at http://seqinspector.cremag.org. When using seqinspector in future studies, please cite this paper.

## Additional files

Additional file 1:
**List of genes upregulated by dexamethason.** (XLS 6 kb) Additional file 2:
**List of  SP1 binding sites in GM12878 human lymphoblastoid cells. **(XLS 92 kb) Additional file 3:
**Lists of astroglia and neuron specific genes. **(XLS 63 kb) Additional file 4:
**List of genes regulated in ruptured intracranial aneurysm. **(XLS 25 kb) Additional file 5:
**Five example sets of genes used for comparison of seqinspector to other tools.** (XLS 33 kb)

## Abbreviations

ChIP-seq: Chromatin immunoprecipitation and next-generation DNA sequencing
ENCODE: Encyclopedia of DNA Elements Consortium
GR: Glucocorticoid receptor
